# Results and Present Position of Radium Therapy in Cancer of Uterus

**Published:** 1916-09

**Authors:** 


### THE RADIUM DEPARTMENT.

Under the Editorial Supervision of DR. EDWARD HOLMAN SKINNER, of Kansas City, Missouri, Assisted by DR, J. M. MARTIN, of Dallas, Texas.

---

It is proposed to present at frequent intervals items of interest upon radium in the form of original articles, collective abstracts, excerpts and editorials.

An attempt will be made to bring authoritative facts before our medical friends which will help them to advise their patients when radium therapy is indicated and when radium is useless.

---

### Results and Present Position of Radium Therapy in Cancer of Uterus.

---

The New York physician, Abbé, first used radium for uterine cancer, but there was really no effective deep radium therapy until 1907, when Dominici extended the principle of filtration to radium. The first authors to report an abundant and convincing material were the pupils of Dominici, Chéron and Rubens Duval; in 1910 they began to use strong filtration and large doses of radium, and in 1911 they reported the first case of a far advanced, inoperable carcinoma of the uterus, shown by later anatomical examination to be completely healed.

In May, 1913, they reported 158 cases, 7 of them operable and the rest inoperable. Radium treatment caused retrogression of the tumor in 155 cases, which was very marked in degree in 93; in 46 there was temporary clinical and subjective recovery, which lasted over a year in 22, and over four years in 1; in 12 cases, inoperable at first, total extirpation was made possible.

Before and soon after the Halle Congress (1913), German authors recommended 150-300 mg. mesothorium with 2-3-mm. lead filters. The results were good so far as temporarily getting rid of the tumors was concerned. Bumm in February, 1914, reported 108 cases, 68 inoperable, 40 operable, 93 temporarily cured, but two-thirds of the cases had not been observed more than six months. Döderlein-Seuffert (April, 1914) reported 52 temporarily cured cases, of which 33 had been operable. Döderlein (March 20, 1915), 12 inoperable cases, which were completely well over a year after treament. Krönig (August, 1914) reported 24 cases mostly treated with radium and mesothorium, free from recurrence, some of them after one, some after two, and some after three years. Weinbrenner temporarily cures 6 out of 20 inoperable cases, and 3 out of 6 operable ones; the rest of the operable cases are still under treatment.

But at the Halle Congress (1913) Bumm reported two cases that had died of the treatment, one from uterine filtration after bladder necrosis; the other from progressive suppuration of the pelvic connective tissue. In February, 1914, he gave a warning against using more than 100 mg. because he had seen cases of progressive hyaline degeneration, strictures and fistula formation, and in July, 1914, he said that radio-active treatment was justified only in superficial carcinomata, and should not be used at all in deep ones, because of the injury to the overlying tissues, in spite of strong filtration. Döderlein's experience was not so bad, but he too saw very painful tenesmus and cicatricial thickening of the wall of the rectum, twice stricture of the rectum, and four times rectovaginal fistulae, probably because of hyaline degeneration of hypertrophied connective tissues.

Of authors who have used 50 to 100 mg., Pinkus reports 16 cases treated, 9 temporarily cured, 1 free from recurrence two years; Klein 40 cases, 11 cured; Schweitzer 26 cases, 7 cured; Adler Schauta 34 cases, 14 cured, or a total of 116 cases with 41 temporarily cured.

It is impossible to say how many of these cases will be cured permanently. Only three cases have been published free from recurrence for more than five years; one by Abbé eight years, and one each by Abbé and Degrais with 6; one case has been published by Wickham and Degrais, one by Degrais and Bellot, and one by Rubens Duval with four years freedom; 2 cases by Bayet, one by Abbé and one by Krönig with three years. These were all inoperable cases. Anatomical examination has also shown the impossibility of complete cure. Only cases are conclusive in which the carcinoma cells have been entirely destroyed; no matter how badly they seem injured, they may recover and proliferate.

In spite of the excellent results of Bumm and his followers, v. Franqué believes that the future radiotherapy of uterine carcinoma will be through the vagina. It is much more effective as shown by his cases. In irradiation through the abdominal wall, a large amount of the Roentgen light is wasted. Therefore it is much more economical to irradiate through the vagina. But Roentgen irradiation through the vagina is very inconvenient for the patient; so the vaginal treatment should be with radioactive substances, and Roentgen irradiation given from ouside for the sake of destroying metastases. This will also be cheaper than the use of the Roentgen rays alone. (Abstracted from article by Franqué, Otton von. Zeit. f. Geburt. v. Gynak., 1915, LXXVII, 244.)

BIBLIOGRAPHY.

Krönig, Muenchen Med. Wochenschr., 1914, LXI, 1715.
Krönig, Deut. Med. Wochenschr., 1913, XXXIX, 1233.
Weinbrenner, Monatsschr. f. Gyn., 1914, XXXIX, 483.
Cheron and Rubens Duval, Gynecologie (Paris), 1913, XVII, 590.
Bayet, J., Med. de Brux., 1914, XIX, 175.
Pinkuss, Berl. Klin. Wochenschr., 1913, L, 1105.
Pinkuss, Berl. Klin. Wochenschr., 1914, LI, 207.
Klein, Muenchen. Med. Wochenschr., 1913, LX, 905.
Döderlein-Seuffert, Muench. Med. Wochenschr., 1914, LXI, 226, 313.
Döderlein-Seuffert, Zentralb. f. Gynak., 1915, 12.
Schauta, Wien. Med. Wochenschr., 1913, LXIII, 2953.
Degrais and Bellot, Clinique (Paris), 1914, IX, 228, 294.
Abbé, Scientific Amer. (Supp.), 1914, LXXVIII, 278.
Bumm, Berl. Klin. Wochenschr., 1914, LI, 1712.
Bumm, Verhandl. d. Berl. Med. Gessells. (1913), 1914, XLIV, 2 Teil, 140.
Bumm, Muench. Med. Wochenschr., 1913, LX, 1697.

